# Editorial: Lipotoxicity, mitotoxicity, and drug targets

**DOI:** 10.3389/fendo.2023.1245111

**Published:** 2023-07-25

**Authors:** Nina Krako Jakovljevic, Neoma T. Boardman, Marina Makrecka-Kuka

**Affiliations:** ^1^ Clinic for Endocrinology, Diabetes and Metabolic Diseases, University Clinical Centre of Serbia, Faculty of Medicine University of Belgrade, Belgrade, Serbia; ^2^ Department Medical Biology, Faculty of Health Sciences, UiT-Arctic University of Norway, Tromsø, Norway; ^3^ Laboratory of Pharmaceutical Pharmacology, Latvian Institute of Organic Synthesis, Riga, Latvia

**Keywords:** lipotoxicity, mitotoxicity, lipids, mitochondria, therapeutics

Lipotoxicity is the dysregulation of the lipid environment leading to the accumulation of harmful lipids that can lead to organelle dysfunction, cellular injury, and cell death. It is often associated with metabolic disease and can occur in many organs, including heart, skeletal muscles, liver, kidney, pancreas, and brain. Although toxic lipids can cause cellular damage through known major mechanisms, it is still unclear how specific lipid classes that directly participate in lipotoxicity contribute to altered cell behavior and mitochondrial bioenergetic functions.

Lipotoxicity, Mitotoxicity, and Drug Targets is a *Frontiers in Endocrinology* Research Topic aimed to identify potential drug targets in the molecular mechanisms behind hyperlipidemia (lipotoxicity)-induced mitochondrial alterations (mitotoxicity). As lipotoxicity is related to altered metabolism and the development of different chronic diseases, such as diabetes, cardiovascular diseases, and neurodegenerative disorders, the goal was to bring forth potential metabolic checkpoints in lipotoxicity-induced cellular dysfunction. This Research Topic puts together different contributions regarding lipotoxicity and mitotoxicity in a broader context of diseases, and also highlights potential therapeutic strategies.

Although it is clear that metabolic health is dependent on a healthy diet and hormonal homeostasis, studies addressing the link between lipidemia and hormones that regulate energy metabolism, such as thyroid hormones, are limited. In a retrospective study, Zhou et al. demonstrated by a cross-sectional analysis the significant correlation between serum palmitic acid (PA) and thyroid dysfunction that was more remarkable in male and obese subjects. This finding highlights the close relationship between lipotoxicity and hypothyroidism, or subclinical hypothyroidism, and points towards potential sex-related differences in lipid metabolism and lipotoxicity progression. Further studies are needed to verify the causal association between PA and thyroid function.

Lipotoxicity is one of the key mechanisms of obesity and plays a role in the development of insulin resistance. Zhao et al. provided a systematic review of the causes and mechanisms of insulin resistance, highlighting that mechanistically, any factor (i.e. lipotoxicity, hypoxia, and inflammation) provoking abnormalities in insulin signaling leads to the development of insulin resistance. The role of the accumulation of different lipid classes in the inhibition of insulin signaling (phosphoinositide 3-kinase - protein kinase B) pathway has previously been widely investigated (1–4). Zhao et al. also explained the role of insulin resistance in the development and progression of metabolism-related chronic diseases targeting different organs. As adipose tissue capacity is overloaded due to obesity, increased free fatty acids (FA) can accumulate ectopically and adversely altered the function of different organs. This mechanism for developing insulin resistance has been modeled in cells (5) where a direct relationship between lipotoxicity and mitotoxicity has been demonstrated. Zhao et al. well summarized available therapeutic strategies for insulin resistance, pointing out the importance of exercise and improved dietary habits. In the broader context of this Research Topic, it might be useful to review the mechanisms of those therapeutic approaches and their effect on mitochondria, as previously described (6, 7). These might be potential modifiers of mitotoxicity and should be taken into consideration in the design of future research studies.

In a mini-review article, Santoro and Feldstein nicely report the role of oxidized lipid species (oxylipins) in insulin resistance and non-alcoholic fatty liver disease (NAFLD) in children. Modern dietary habits, rich in omega-6 polyunsaturated fatty acids (n-6 PUFA) and low in n-3 PUFA, are the main reason for intrahepatic fat accumulation that is often preceded by insulin resistance (8). Moreover, these conditions are also associated with subtle inflammation that provides a favorable environment for the formation of oxylipins. The authors suggest that oxylipins may be a pathogenic link between NAFLD and diabetes and report a clinical study (9) that used pharmacology treatment to reverse oxylipins that improved liver fibrosis and inflammation. They clearly concluded that therapeutic efforts should aim to reduce plasma levels of n-6 PUFA as well as n-6 PUFA intake in order to prevent insulin resistance and NASH in children.

When the supply of FAs exceeds mitochondrial FA oxidation capacity, it can result in the accumulation of FA intermediates, such as long-chain acylcarnitines, diacylglycerol, and ceramides (10, 11). In addition to causing oxidative stress, FA intermediates can inhibit glucose metabolism, and disrupt insulin signaling and oxidative phosphorylation in the mitochondria (8). In agreement, Jansen et al. report a marked reduction in mitochondrial respiration in different organs, under various respiratory conditions, following chronic high-fat feeding in mice. The GLP1 analogue, exenatide, given to high-fat mice attenuated this response in liver mitochondria although this effect was not observed in either adipose tissue or skeletal muscle. This observation is an important reminder of the inter-organ differences in mitochondrial function as well as different inter-organ responses to treatment that may also be important when evaluating effects on whole-body metabolism, weight loss, and insulin sensitivity. As such, where the novel marine Calanus oil was previously shown to be protective in cardiac mitochondria from high-fat mice (12), in the present study Calanus oil treatment did not alter mitochondrial function in any of the organs tested. Jansen et al. speculate that elevated leak respiration in the liver may be preventing lipotoxicity through increased FA oxidation and thus reduce the accumulation of lipid intermediates. This would, in turn, be important for mitochondrial respiratory capacity and homeostasis, and may contribute to an overall anti-obesogenic effect.

Efficient FA oxidation is key to maintaining lipid homeostasis in the cells, and this may be especially important under stress conditions, like limited oxygen during ischemia (13). Peroxisome proliferator-activated receptors (PPAR) are regulators of lipid metabolism and can modulate the expression of target genes related to metabolism and inflammation. Papatheodorou et al. demonstrate a central role for PPARB/d under cardiac stress (ischemia-reperfusion), suggesting that upregulation of FA oxidation during ischemia-reperfusion is cardioprotective. Importantly PPARB/d activation also induces NFR2/PGC1α and antioxidant pathways suggesting a broader role for the regulation of lipid homeostasis. Future studies should address remaining questions that include investigating a direct link between lipid availability on mitochondrial structure, a detailed examination of mitochondrial integrity such as the membrane composition, cristae structures, and formation of respiratory supercomplexes, and how different lipids affect mitochondrial functionality under physiological and stress conditions.

## Author contributions

NKJ, NB and MM-K conceived the original draft, wrote the original manuscript and edited final manuscript. All authors contributed to the article and approved the submitted version.
